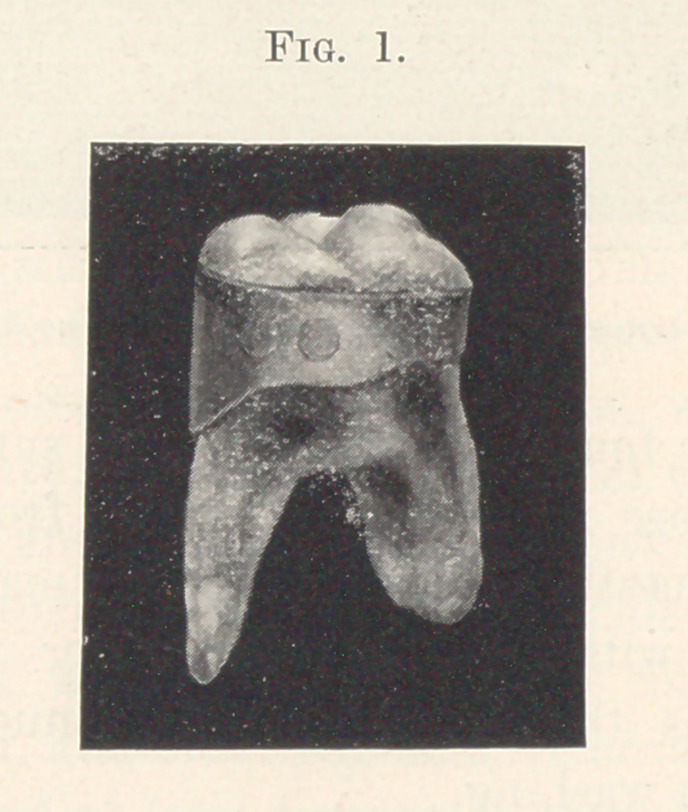# A Simple, Efficient Crown for the Posterior Teeth

**Published:** 1905-07

**Authors:** Allison R. Lawshe

**Affiliations:** Trenton, N. J.


### A SIMPLE, EFFICIENT CROWN FOR THE POSTERIOR TEETH.

BY ALLISON R. LAWSHE, D.D.S., TRENTON, N. J.

HAVING found much satisfaction from the use of an easily made crown for badly broken down second and third molar teeth, and for first molar teeth not exposed to view, I have thought that others may also find it useful.

Devitalize the pulp, if living, and prepare the root-canal as usual. Grind down the walls, if any remain, half way, and slope to the gum margin. Prepare a well-fitting band of gold, leaving space for cusps, as is usual with all-gold crowns, and make two countersunk holes on the buccal and two on the lingual side. Now coat the inside lower edge of this band with a layer of gutta-percha,

FIG. 1.

that it may make a perfect fit when pressed to place, and after preparing good, substantial undercuts in the pulp-chamber, or the openings of the root-canals, warm and adjust it, wash the root-stump with alcohol, and carefully and thoroughly pack amalgam into the undercuts and all spaces between stump and band, filling the entire band and building cusps above it. The patient can then be instructed to bite, and the building and carving of the cusps continued until a perfect articulation is secured, when the crown may be considered finished (Fig. 1). A tooth near by should then be washed with chloroform, dried, and a ball of tough gutta-percha fastened thereto to prevent the possibility of damage to the crown by the occluding teeth before the amalgam has set.

Besides being easily made, this crown has the merit of a perfect articulation and comparative ease of adjustment in mouths where an excessive flow of saliva would render the placement of the ordinary gold cap on a thoroughly dried root troublesome and uncertain. It is, moreover, strong and durable.

## Figures and Tables

**Fig. 1. f1:**